# Zinc Transporter 3 (Znt3) as an Active Substance in the Enteric Nervous System of the Porcine Esophagus

**DOI:** 10.1007/s12031-016-0854-0

**Published:** 2016-10-30

**Authors:** Joanna Wojtkiewicz, Krystyna Makowska, Ewa Bejer-Olenska, Sławomir Gonkowski

**Affiliations:** 1 Department of Pathophysiology, Faculty of Medical Sciences, Warszawska Str. 30, 10-082 Olsztyn, Poland; 2 Department of Clinical Physiology, Faculty of Veterinary Medicine, University of Warmia and Mazury, Oczapowskiego Str. 13, 10-718 Olsztyn, Poland

**Keywords:** Zinc transporters, Esophagus, Enteric nervous system

## Abstract

Zinc transporter 3 (ZnT3), a member of the SLC 30 zinc transporter family, is involved in the transport of zinc ions from the cytoplasm into synaptic vesicles or intracellular organelles. The aim of the present study was to investigate for the first time the percentage of ZnT3-like immunoreactive (ZnT3-LI) neurons in the enteric nervous system (ENS) of the porcine esophagus and denotation of their neurochemical coding. Routine double- and triple-immunofluorescence labeling of cervical, thoracic, and abdominal fragments of esophagus for ZnT3 with protein gene product (PGP 9.5; used as pan-neuronal marker), nitric oxide synthase (NOS), somatostatin, vasoactive intestinal peptide (VIP), vesicular acetylcholine transporter (VAChT), neuropeptide Y (NPY), and galanin (GAL) was performed. The percentage of ZnT3-LI neurons in myenteric ganglia amounted to 50.2 ± 4.7, 63.4 ± 8.3, and 77.1 ± 1.1 % of all PGP 9.5-like immunoreactive neuronal cells in cervical, thoracic, and abdominal esophagus, respectively. In submucous ganglia, these values in particular parts of esophagus amounted to 46.3 ± 6.3, 81.0 ± 8.1, and 74.4 ± 4.4 %. Znt3 co-localized mainly with VAChT, NPY, GAL, NOS, and VIP, but the degree of co-localization depended on the “kind” of enteric ganglia and part of esophagus studied. The obtained results suggest that both ZnT3 and zinc ions may play important and various roles in the neuronal regulation of esophageal functions.

## Introduction

The enteric nervous system (ENS) is made up of millions of neuronal cells located in the wall of the gastrointestinal (GI) tract from esophagus to anus (Furness et al. 2014). Its organization depends on animal species and the part of the GI tract (Timmermans et al. 1997; Brown and Timmermans 2004). In esophagus and stomach, the ENS consists of the following two kinds of intramural ganglia: myenteric ganglia (MG), which are connected to each other by density network of nerves and create myenteric plexus located between the longitudinal and circular muscle layers, as well as submucous ganglia (SG), which not form a plexus and situated near the lamina propria of the mucosal layer (Teixeira et al. 2001; Zacharko-Siembida and Arciszewski 2014; Chiocchetti et al. 2015; Rekawek et al. 2015). The same types of enteric ganglia are present in small and large intestine of rodents, but contrary to esophagus and stomach, also submucous ganglia form plexus (Paulino et al. 2011). In small and large intestine of big mammals (for example in pig), submucous plexus is divided into outer submucous plexus located near internal side of the circular muscle layer and inner submucous plexus—between the muscularis mucosa and lamina propria (Brown and Timmermans 2004; Gonkowski et al. 2009a, 2012a).

In spite of the fact that ENS receives signals from extrinsic parasympathetic and sympathetic nervous structures (Wojtkiewicz et al. 2013), it is characterized by significant autonomy and able to fully function in the absence of central input. The ENS takes part in the regulation of all functions of the GI tract, such as intestinal motility, excretive activity of the mucosal layer, fluid exchange between the wall of stomach and gut and their lumen, and local blood flow (Furness et al. 2014). Moreover, it is known that enteric neurons can also play important functions under various pathological stimuli and take part in adaptive, regenerative, and/or neuroprotective processes during intestinal and extra-intestinal diseases (Gonkowski et al. 2003; Vasina et al. 2006; Gonkowski 2013).

All above-mentioned functions of the ENS are realized with a range spectrum of neuronal active substances, which most often can play roles of neuromediators and/or neuromodulators. Several dozen such substances have been described in neuronal cells and nerves within the ENS. Besides acetylcholine—typical neuromediator for parasympathetic nervous system, also other active substances have been described within enteric neurons. The most important of these substances include vasoactive intestinal polypeptide (VIP), somatostatin (SOM), substance P (SP), pituitary adenylate cyclase-activating peptide (PACAP), nitric oxide (NO), galanin (GAL), and many others (Vasina et al. 2006; Furness et al. 2014). One of substances which functions within the enteric nervous system remain still obscure is zinc transporter 3 (ZnT3) (Wojtkiewicz et al. 2012a, b).

ZnT3 is a member of SLC 30 family zinc transporters, which enable the transport of hydrophilic zinc ions from the cytoplasm into synaptic vesicles, intracellular organelles, or to the outside of the cell. Ten members of ZnT peptide family (marked by abbreviation ZnT1–ZnT10) have been identified in various tissues of mammals (Palmiter and Huang 2004). All these transporters are constructed of six transmembrane domains and loop of amino acid chain rich in histidine, which is the place zinc ions bind. In all ZnT transporters, only ZnT3 is closely associated with neuronal cells, which takes part in conduction of impulses by the transport of zinc ions to synaptic vesicle (Palmiter et al. 1996), although the newest studies describe the presence of ZnT3 also in pancreatic beta cells (Smidt et al. 2016).

ZnT3 has been described in different regions of the central and peripheral nervous system (Wenzel et al. 1997; Wang et al. 2002, 2005; Kaneko et al. 2015). First of all, Znt3 is considered to be a marker of zinc-enriched (ZEN) terminals within brain, spinal cord, and superior cervical ganglion (Jo et al. 2000; Wenzel et al. 1997). These nerves, and indeed ZnT3, show inhibitory effects and take part in sensory conduction and excretive function, as well as play some, not fully explained, roles during pathological processes, such as epilepsy, cerebral ischemia, and amyotrophic lateral sclerosis (Takeda 2000; Danscher et al. 2001; Molnar and Nadler 2001; Kaneko et al. 2015).

ZnT3 has been also observed in the ENS, but it should be pointed out that the knowledge about distribution of ZnT3 in this part of nervous system is very scanty. Namely, this substance has been described in human large and porcine small intestine, and its functions in neuronal processes within the GI tract are completely unknown (Gonkowski et al. 2009b; Wojtkiewicz et al. 2012a, b). So, the present study describes for the first time the localization and chemical coding of ZnT3-like immunoreactive neurons within the enteric nervous system of porcine esophagus and may be the introduction for further investigations on exact functions of Znt3 within the digestive system.

## Materials and Methods

The present investigation was performed on six immature female pigs of the Large White Polish breed (approximately 8 weeks old). Animals were kept under standard laboratory conditions, and all experimental procedures were made following the instructions of the Local Ethical Committee in Olsztyn (Poland), with special attention paid to minimizing any stress reaction during investigation.

After adaptive period in laboratory (3 days), animals were pre-treated with Stressnil (Janssen, Belgium, 75 μl/kg of body weight, i.m.) 15 min before the euthanasia by an overdose of sodium thiopental (Thiopental, Sandoz, Kundl-Rakúsko, Austria) given intravenously. Then, pigs were perfused transcardially with 4 % buffered paraformaldehyde prepared ex tempore. The selfsame parts (ca. 1 cm long) of cervical, thoracic, and abdominal esophagus were collected from all animals studied; post-fixed by immersion in the same fixative for 30 min; rinsed in phosphate buffer for several hours; stored in 18 % sucrose until sectioning (at least 10 days); and finally, cut into 10-μm-thick cryostat sections. These sections were subjected to standard double- and triple-labeling immunofluorescence as described previously by Gonkowski et al. (2012b) and Wojtkiewicz et al. (2012a). In essence, the immunofluorescence procedure has been made as follows: after air-drying at room temperature (RT) for 45 min, sections of esophagus were incubated with a blocking solution containing 10 % normal goat serum, 0.1 % bovine serum albumin, 0.01 % NaN3, Triton X-100, and thimerozal in PBS for 1 h (RT). Then, they were incubated (overnight; RT, in a humid chamber) with a mixture of two (in double-immunofluorescence technique) or three (in triple-immunofluorescence technique) antibodies raised in different species and directed towards zinc transporter 3 and one of the other selected neuronal active substance, i.e., protein gene product 9.5 (PGP 9.5; used here as pan-neuronal marker), vesicular acetylcholine transporter (VAChT; used here as marker of cholinergic neurons), neuropeptide Y (NPY), vasoactive intestinal polypeptide (VIP), somatostatin (SOM), galanin (GAL), or nitric oxide synthase (NOS)—a marker of nitrergic processes, raised in different species (the precise specification of anti-sera is presented in Table 1). Complexes of primary antibodies bound to appropriate antigens were visualized by incubation (1 h, RT) with species-specific secondary anti-sera conjugated to FITC or biotin, and the latter antibodies were then visualized by a streptavidin-CY3 complex (1 h, RT). Each step of immunolabeling was followed by rinsing the sections with PBS (3 × 10 min, pH 7.4). Standard controls, i.e., pre-absorption of the neuropeptide anti-sera with appropriate antigen, omission, and replacement of primary anti-sera by non-immune sera, were performed to test the antibodies and specificity of the method.

**Table 1:** Specification of immune reagents vs. zinc transporter 3

Primary antibody				
Antisera	Code	Host species	Dilution	Supplier
PGP 9.5	7863–2004	Mouse	1:2000	Biogenesis Inc., UK; www.biogenesis.co.uk
ZnT3	–	Rabbit	1:600	Gift prof. Palmiter, USA
GAL	T-5036	Guinea pig	1:1000	Peninsula Labs, USA; see Bachem AG; www.bachem.com
NOS	N2280	Mouse	1: 2000	Sigma, USA; www.sigma-aldrich.com
NPY	NZ1115	Rat	1:300	Biomol Research Laboratories Inc., USA
SOM	8330–0009	Rat	1: 100	Biogenesis Inc., UK; www.biogenesis.co.uk
VAChT	H-V007	Goat	1: 2000	Phoenix Pharmaceuticals Inc., USA; www.phoenixpeptide.com
VIP	9535–0504	Mouse	1: 2000	Biogenesis Inc., UK; www.biogenesis.co.uk
Secondary antibodies				
Reagent			Dilution	Supplier
FITC-conjugated donkey-anti-mouse IgG (H + L)			1:800	Jackson, 715–095-151
FITC-conjugated donkey-anti-rat IgG (H + L)			1:800	Jackson, 712–095-153
FITC-conjugated donkey-anti-guinea pig IgG (H + L)			1:1000	Jackson, 706–095-148
FITC-conjugated donkey-anti-goat IgG (H + L)			1:1000	Jackson, 705–096-147
Biotinylated goat anti-rabbit immunoglobulins			1:1000	DAKO, E 0432
Biotin-conjugated F(*ab*)′ fragment of affinity-purified anti-rabbit IgG (H + L)			1:1000	BioTrend, 711–1622
AMCA-conjugated donkey-anti-mouse IgG (H + L)			1:50	Jackson, 715–155-151
AMCA-conjugated donkey-anti-rat IgG (H + L)			1:50	Jackson, 715–155-153
AMCA-conjugated donkey-anti-goat IgG (H + L)			1:50	Jackson, 705–156-147
CY3-conjugated streptavidin			1:9000	Jackson, 016–160-084

*PGP 9.5* pan-neuronal marker, *ZnT3* zinc transporter 3, *NOS* nitric oxide synthase, *VIP* vasoactive intestinal peptide, *SOM* somatostatin, *VAChT* vesicular acetylcholine transporter, *NPY* neuropeptide Y, *GAL* galanin, *CGRP* calcitonin gene-related peptide, *FITC* fluorescein isothiocyanate, *AMCA* 7-amino-4-methylcoumarin-3-acetic acid, *H* heavy chain, *L* light chain

To evaluate the percentage of populations ZnT3-like immunoreactive neurons, at least 700 PGP 9.5-labeled cell bodies in myenteric (MG) and submucous ganglia (SG) of esophagus of each studied animal were examined. Moreover, to determine the percentages of co-localization of ZnT3 with other substances studied, at least 500 ZnT3-positive cell bodies in particular types of enteric ganglia were examined for immunoreactivity to the particular substances investigated. In these studies, ZnT3-positive neurons were considered as representing 100 % for all combinations. The same method was used to establish the percentage of ZnT3-positive cells with reference to neuronal populations immunoreactive to GAL, NOS, NPY, SOM, VAChT, and VIP, but in this case, the numbers of cells immunoreactive to particular substances studied were considered as 100 %.

Double-labeled perikarya (only neurons with clearly visible nucleus were included) were determined under an Olympus BX51 microscope equipped with epi-fluorescence and appropriate filter sets, pooled, and presented as mean ± SEM. To prevent double counting of SP-LI neurons, the sections were located at least 100 μm apart. All pictures were captured by a digital camera connected to a PC. Statistical analysis was carried out with Student’s *t* test (GraphPad Prism v. 6.0; GraphPad Software Inc., San Diego, CA, USA). The differences were considered statistically significant at *p* ≤ 0.05.

## Results

### The Number of ZnT3^+^ Neurons in the Porcine Esophagus

**Fig. 1:**
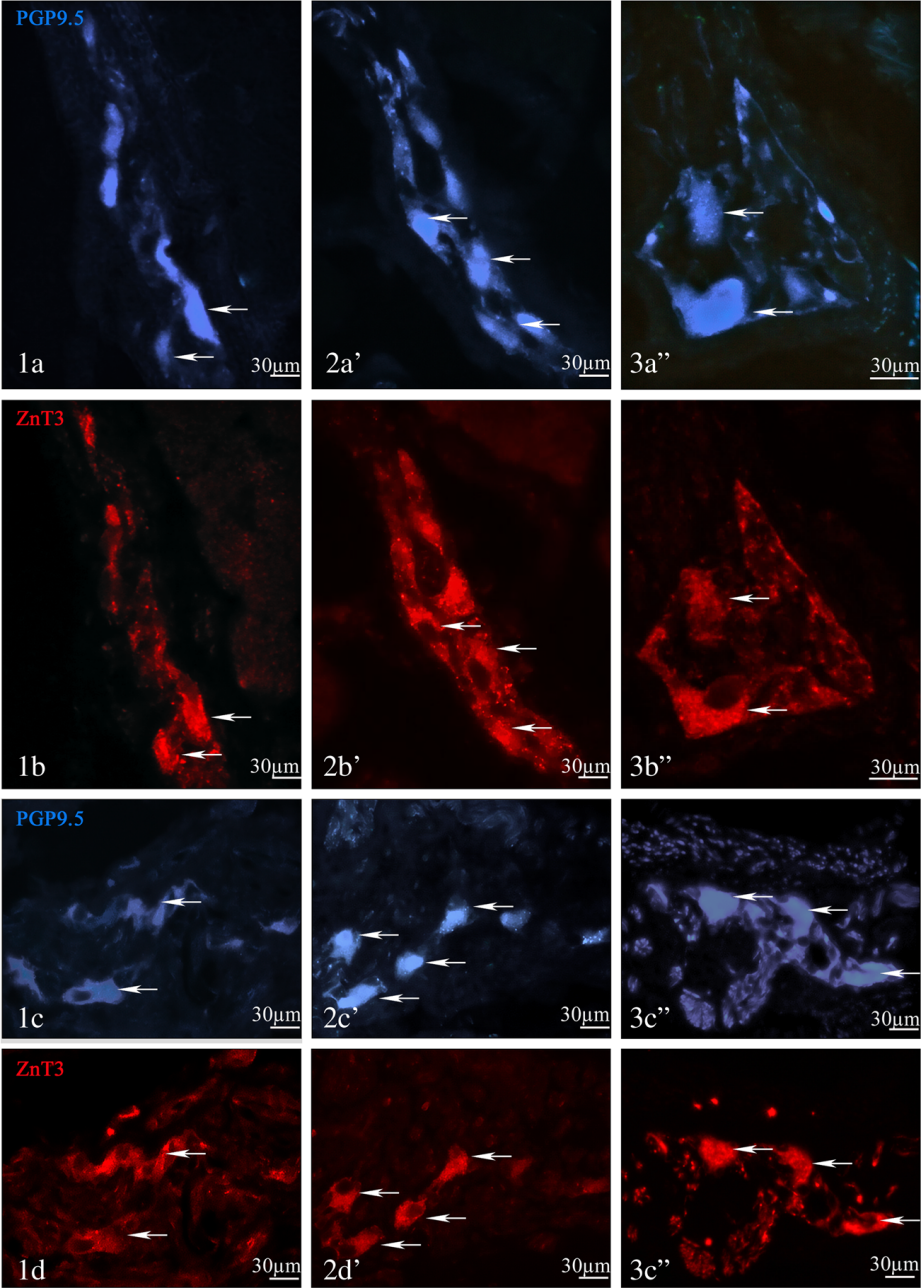
Representative images of various fragment of the porcine esophagus for PGP9.5 and ZnT3: the cervical part—mesenteric ganglia (MP) (*1a*, *1b*), and submucosal ganglia (SG) (***1*** *c*, *1d*); the thoracic part—MP (*2a'*, *2b'*) and SG (***2*** *c'*, *2d'*); and the abdominal part—MP (*3a"*, *3b"*) and SG (*3c"*, *3d"*)

During the present investigation, neuronal cells immunoreactive to ZnT3 were observed in myenteric and submucous enteric ganglia within all fragments of porcine esophagus studied (Table 2 and Fig. 1). The number of such cells was relatively considerable in both “kinds” of ganglia and clearly depends on the fragment of esophagus. Within myenteric ganglia of cervical esophagus, the percentage of ZnT3-positive neuronal cells amounted to 50.2 ± 4.7 % of all PGP 9.5-like immunoreactive neurons. In posterior parts of esophagus, these values were even higher and amounted to 63.4 ± 8.3 and 77.1 ± 1.1 % in thoracic and abdominal esophagus, respectively. Numerous population of ZnT3-positive neuronal cell was also noted in submucous ganglia, where the percentage of these neurons amounted to 46.3 ± 6.3, 81.0 ± 8.1, and 74.4 ± 4.4 % of PGP 9.5-LI cells in cervical, thoracic, and abdominal esophagus, respectively.

**Table 2:** Neurochemical characterization of zinc transporter 3-like immunoreactive (ZnT3^+^) neurons in the enteric ganglia of the porcine esophagus

	Myenteric ganglia	Submucous ganglia
Cervical esophagus		
PGP 9.5^+^/ZnT3^+a^	50.2 ± 4.7	46.3 ± 6.3
ZnT3^+^/GAL^+^	85.2 ± 1.8	31.8 ± 2.6
ZnT3^+^/NOS^+^	45.5 ± 3.7	60.8 ± 7.9
ZnT3^+^/NPY^+^	87.3 ± 6.0	2.8 ± 0.6
ZnT3^+^/SOM^+^	0	0
ZnT3^+^/VAChT^+^	87.9 ± 2.7	80.3 ± 3.0
ZnT3^+^/VIP^+^	60.6 ± 2.0	66.2 ± 1.8
Thoracic esophagus		
PGP 9.5^+^/ZnT3^+a^	63.4 ± 8.3	81.0 ± 8.1
ZnT3^+^/GAL^+^	57.0 ± 4.2	29.7 ± 13.0
ZnT3^+^/NOS^+^	62.3 ± 8.0	30.4 ± 4.6
ZnT3^+^/NPY^+^	77.7 ± 7.5	6.0 ± 3.3
ZnT3^+^/SOM^+^	0	0
ZnT3^+^/VAChT^+^	54.9 ± 3.8	78.0 ± 10.5
ZnT3^+^/VIP^+^	40.4 ± 1.4	6.3 ± 1.5
Abdominal esophagus		
PGP 9.5^+^/ZnT3^+a^	77.7 ± 1.1	74.4 ± 4.4
ZnT3^+^/GAL^+^	28.8 ± 2.0	42.8 ± 5.0
ZnT3^+^/NOS^+^	79.0 ± 6.0	79.0 ± 5.7
ZnT3^+^/NPY^+^	44.9 ± 8.2	28.8 ± 3.2
ZnT3^+^/SOM^+^	0	0
ZnT3^+^/VAChT^+^	25.1 ± 5.0	23.23 ± 1.5
ZnT3^+^/VIP^+^	87.2 ± 7.6	79.3 ± 8.1

Note that PGP 9.5 is a pan-neuronal marker that marks all neurons in the tissue, and so, PGP^+^/ZnT3^+^ cells illustrate the percentage of ZnT3-positive neurons. In case of co-localization of ZnT3 with other neurochemical factors, ZnT3-positive neurons were considered as representing 100 % for all combinations with other neurotransmitters, and so, all the values presented are percentages (means ± SEM) of ZnT3^+^ neurons

^a^The percentage of ZnT3-positive neurons with reference to all cells immunoreactive to PGP 9.5 (used here as pan-neuronal marker; number of PGP 9.5-positive cells = 100 %)

### The Co-Localization of ZnT3 with Other Active Substances in the ENS of Porcine Esophagus

A broad spectrum of active substances was noted in ZnT3-positive neuronal cells of the esophageal ENS during the present study, and the degree of co-localization of ZnT3 with particular substances depends on both “kind” of enteric ganglion, as well as the fragment of esophagus (Table 2).

### Cervical Esophagus

**Fig. 2:**
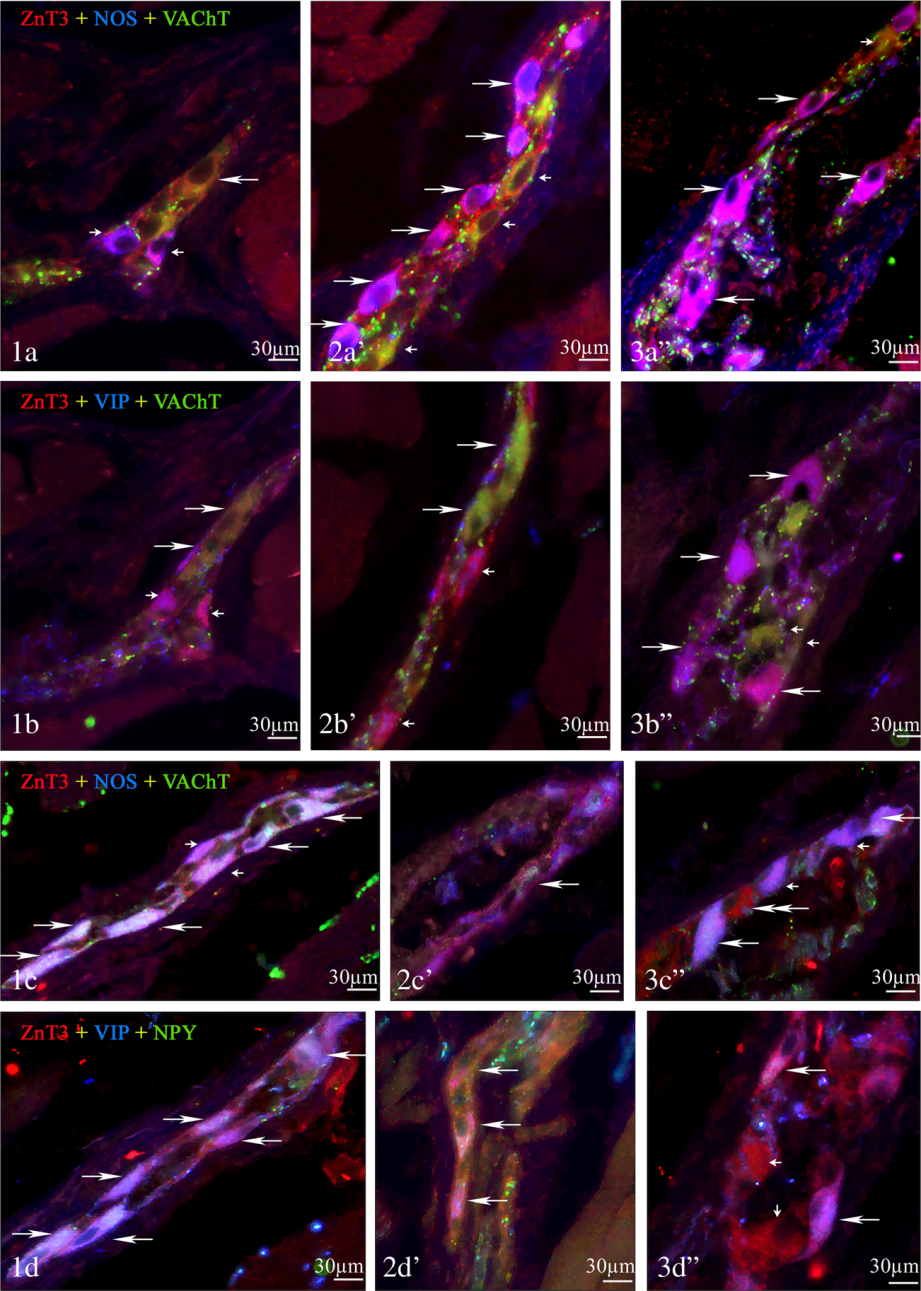
Representative images of ZnT3^+^ neurons located in the various part of porcine esophagus, myenteric ganglia. All images are composites of merged images taken separately from *blue*, *red*, and *green* fluorescent channels. (*1*) The cervical part—(*1a*) ZnT3^+^/NOS^−^/VAChT^+^ neuron is indicated with *arrow*, and ZnT3^+^/NOS^+^/VAChT^−^ neurons are indicated with *small arrows*; (*1b*) ZnT3^+^/VIP^−^/VAChT^+^ neurons are indicated with *arrows*, and ZnT3^+^/VIP^+^/VAChT^−^ neurons are indicated with *small arrows*; (*1c*) ZnT3^+^/NOS^+^/NPY^+^ neurons are indicated with *arrows*, and ZnT3^+^/NOS^+^/NPY^−^ neurons are indicated with *small arrows*; and (*1d*) ZnT3^+^/VIP^+^/NPY^+^ neurons are indicated with *arrows*. (*2*) The thoracic part—(*2a*′) ZnT3^+^/NOS^+^/VAChT^−^ neurons are indicated with *arrows*, and ZnT3^+^/NOS^−^/VAChT^+^ neurons are indicated with *small arrows*; (*2b′*) ZnT3^+^/VIP^+^/VAChT^−^ neurons are indicated with *arrows*, and ZnT3^+^/VIP^−^/VAChT^−^ neurons are indicated with *small arrows*; (*2c′*) ZnT3^+^/NOS^−^/NPY^+^ neuron is indicated with *arrow*, and ZnT3^+^/NOS^+^/NPY^−^ neuron is indicated with *small arrow*; and (*2d′*) ZnT3^+^/VIP^+^/NPY^−^ neurons are indicated with *arrows*. (*3*) The abdominal part—(*3a*″) ZnT3^+^/NOS^+^/VAChT^−^ neurons are indicated with *arrows*, and ZnT3^+^/NOS^−^/VAChT^+^ neuron is indicated with *small head*; (*3b*″) ZnT3^+^/VIP^+^/VAChT^−^ neurons are indicated with *arrows*, and ZnT3^+^/VIP^−^/VAChT^+^ neurons are indicated with *small arrows*; (*3c*″) ZnT3^+^/NOS^+^/NPY^+^ neurons are indicated with *arrows*, ZnT3^+^/NOS^+^/NPY^−^ neurons are indicated with *small arrows*, and ZnT3^+^/NOS^−^/NPY^−^ neuron is indicated with *double-headed arrow*; and (*3d*″) ZnT3^+^/VIP^+^/NPY^+^ neurons are indicated with *arrows*, and ZnT3^+^/VIP^−^/NPY^−^ neurons are indicated with *small arrows*

In myenteric ganglia of cervical esophagus (Fig. 2 (1)), the up to 87.9 ± 2.7 % of all ZnT3-LI neurons also showed expression of VAChT. Similar percentage of cells immunoreactive to ZnT3were also NPY- and/or GAL-positive (87.3 ± 6.0 and 85.2 ± 1.8 %, respectively). A slightly lower degree of co-localization ZnT3 with VIP and/or NOS was noted. The percentages of such neuronal populations amounted to 60.6 ± 2.0 % (ZnT3^+^/VIP^+^) and 45.5 ± 3.7 % (ZnT3^+^/NOS^+^). In turn, SOM was no observed in ZnT3^+^ neurons in myenteric ganglia of cervical porcine esophagus during the present study (Table 2).

**Fig. 3:**
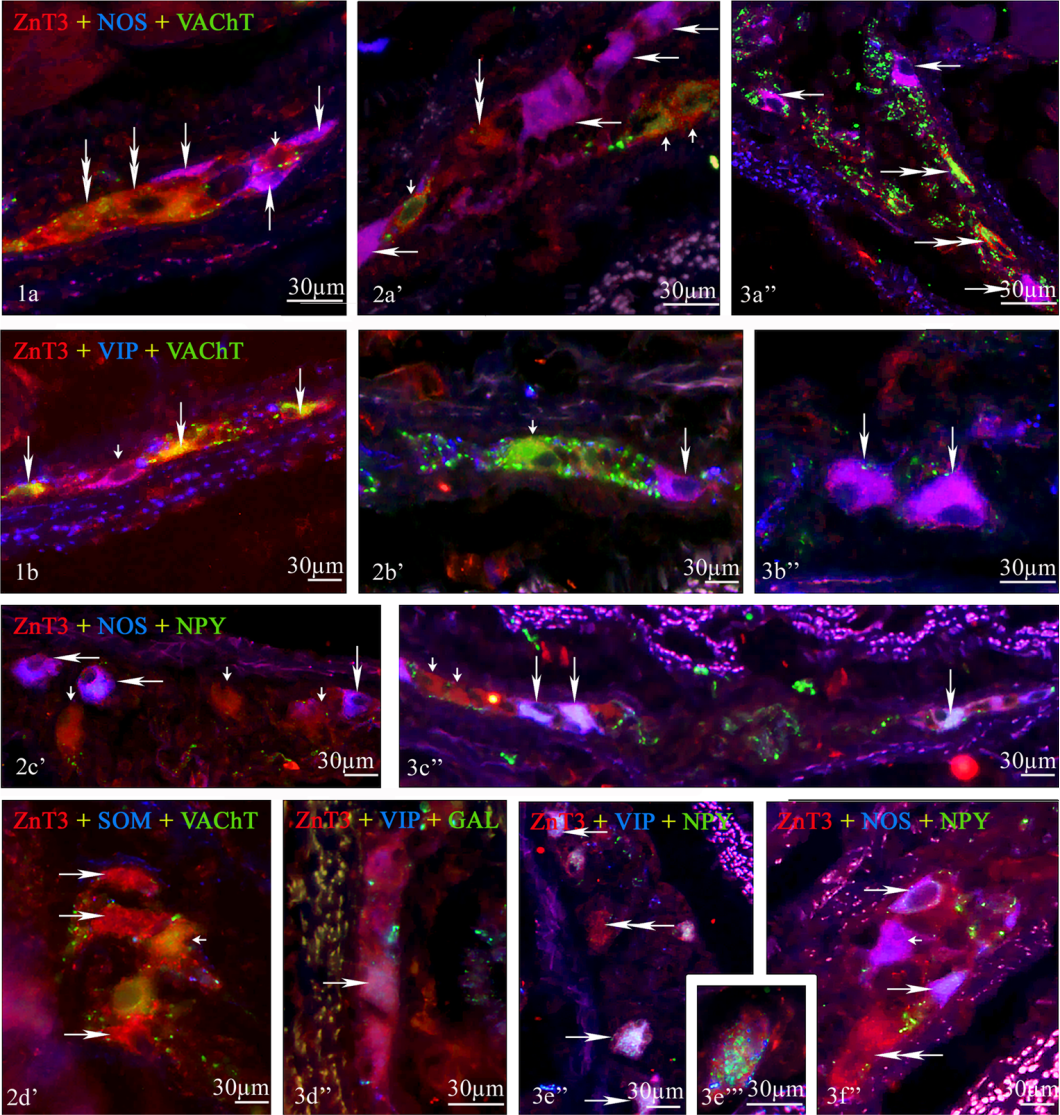
Representative images of ZnT3^+^ neurons located in the various part of porcine esophagus, submucous ganglia. All images are composites of merged images taken separately from *blue*, *red*, and *green* fluorescent channels. (*1*) The cervical part—(*1a*) ZnT3^+^/NOS^+^/VAChT^−^ neurons are indicated with *arrows*, ZnT3^+^/NOS^−^/VAChT^+^ cells are indicated with *small arrows*, and ZnT3^+^/NOS^−^/VAChT^−^ neuron is indicated with *double-headed arrow* and (*1b*) ZnT3^+^/VIP^−^/VAChT^+^ neurons are indicated with *arrows*, and ZnT3^+^/VIP^+^/VAChT^−^ neuron is indicated with *small arrow*. (*2*) The thoracic part—(*2a*′) ZnT3^+^/NOS^+^/VAChT^−^ neurons are indicated with *arrows*, ZnT3^+^/NOS^−^/VAChT^+^ neurons are indicated with *small arrows*, and ZnT3^+^/NOS^−^/VAChT^−^ neuron is indicated with *double-headed arrow*; (*2b*′) ZnT3^+^/VIP^+^/VAChT^−^ neuron is indicated with *arrow*, and ZnT3^+^/VIP^−^/VAChT^+^ neuron is indicated with *small arrow*; (*2c*′) ZnT3^+^/NOS^+^/NPY^−^ neurons are indicated with *arrows*, and ZnT3^+^/NOS^−^/NPY^+^ neurons are indicated with *small arrows*; and (*3d*′) ZnT3^+^/SOM^−^/VAChT^−^ cells are indicated with *arrows*, and ZnT3^+^/SOM^−^/VAChT^+^ neuron is indicated with *small arrow*. (*3*) The abdominal part—(*3a*″) ZnT3^+^/NOS^+^/VAChT^−^ neurons are indicated with *arrows*, ZnT3^+^/NOS^−^/VAChT^+^ neurons are indicated with *small arrows*, and ZnT3^+^/NOS^−^/VAChT^−^ neuron is indicated with *double-headed arrow*; (*3b*″) ZnT3^+^/VIP^+^/VAChT^−^ neurons are indicated with *arrows*; (*3c*″) ZnT3^+^/NOS^+^/NPY^+^ neurons are indicated with *arrows*, ZnT3^+^/NOS^+^/NPY^−^ neuron is indicated with *small arrow*, and ZnT3^+^/NOS^−^/NPY^−^ is indicated with *double-headed arrow*; (*3d*″) ZnT3^+^/VIP^+^/GAL^+^ neurons are indicated with *arrows*; (*3e*″) ZnT3^+^/VIP^+^/NPY^+^ neurons are indicated with *arrows*, and ZnT3^+^/NOS^−^/NPY^−^ is indicated with *double-headed arrow*; and (*3f*″) ZnT3^+^/NOS^+^/GAL^+^ neurons are indicated with *arrows*, ZnT3^+^/NOS^+^/GAL^−^ neuron is indicated with *small arrow*, and ZnT3^+^/NOS^+^/GAL^−^ neuron is indicated with *small arrow*

In submucous ganglia of cervical esophagus (Fig. 3 (1)), the chemical coding of Znt3-positive neurons differed from those, which noted within myenteric ganglia (Table 2). The greatest differences concerned the co-localization of ZnT3 with NPY, GAL, and/or NOS. In SG, the percentage of ZnT3^+^/NPY^+^ and ZnT3^+^/GAL^+^ cells were lower than in myenteric ganglia, which was particularly visible in the event of NPY. Namely, these values amounted to 2.8 ± 0.6 and 31.8 ± 2.6 %, respectively. In turn, the number of neurons simultaneously immunoreactive to Znt3 and NOS was higher than within MG and stood at 60.8 ± 7.9 %. The level of co-localization of ZnT3 with other substances studied in SG was similar to those observed in MG.

### Thoracic Esophagus

In myenteric ganglia of thoracic esophagus (Fig. 2 (2)), the most of ZnT3-positive cells were also immunoreactive to NPY (77.7 ± 7.5 % of all ZnT3^+^ neurons), NOS (62.3 ± 8.0 %), and/or GAL (57.0 ± 4.2 %). In turn, the degree of co-localization of Znt3 with VAChT and/or VIP was markedly lower than within cervical esophagus and amounted to 54.9 ± 3.8 and 40.4 ± 1.4 %, respectively. Within submucous ganglia (Fig. 3 (2)), the number of ZnT3^+^/VAChT^+^ neuronal cells was higher than in myenteric ganglia and amounted to 78.0 ± 10.5 % of all neurons immunopositive to ZnT3. Other substances studied were less often observed in ZnT3-like immunoreactive cells. The percentage of ZnT3^+^/NOS^+^ and ZnT3^+^/GAL^+^ neurons reached 30.4 ± 4.6 and 28.7 ± 13.0 %, respectively. In turn, the number of neurons, in which the co-localization of ZnT3 with VIP and ZnT3 with NPY was noted, fluctuated around 6 % of all ZnT3-LI neurons (6.3 ± 1.5 and 6.0 ± 3.3 %, respectively). Moreover, SOM was no observed in ZnT3-positive neurons in the neither myenteric nor submucous ganglia of thoracic esophagus.

### Abdominal Esophagus

The most of ZnT3-positive neuronal cells in myenteric ganglia (Fig. 2 (3)) were also immunoreactive to VIP (87.2 ± 7.6 % of all ZnT3^+^ neurons) and NOS (79.0 ± 6.0 %). A slightly less large population (44.9 ± 8.2 %) was cell immunoreactive simultaneously to ZnT3 and NPY. In turn, VAChT and GAL were noted in about a quarter of all Znt3-positive myenteric neurons (25.1 ± 5.0 and 28.8 ± 2.0 %, respectively).

Within submucous ganglia (Fig. 3 (3)), the degree of co-localization of ZnT3 with NOS and/or VAChT was similar to those, which was observed in myenteric ganglia. These values amounted to 79.0 ± 5.7 and 23.23 ± 1.5 % of all ZnT3-LI neuronal cells. The number of ZnT3^+^/VIP^+^ and ZnT3^+^/NPY^+^ was lower than in MG (79.3 ± 8.1 and 28.8 ± 3.2 %, respectively). The percentage of neurons immunoreactive simultaneously to ZnT3 and GAL amounted to 42.8 ± 5.0 % of all ZnT3^+^ and was higher than the percentage of such type of cells within MG. Moreover, the co-localization of ZnT3 and SOM was no observed in the enteric nervous system of abdominal esophagus (Table 2).

Moreover, ZnT3-positive perikarya were comprised a large percentage of neurons immunoreactive to the majority of active substances studied, and the biggest degree of co-localization was observed in VIP^+^ neurons in the cervical esophagus, where all cells immunoreactive to VIP were also ZnT3-LI (Table 3).

**Table 3:** The percentage (means ± SEM) of zinc transporter 3-like immunoreactive (ZnT3^+^) perikarya in neuronal populations immunoreactive to particular active substances studied

	Myenteric ganglia	Submucous ganglia
Cervical esophagus		
GAL^+^/ZnT3^+^	93.0 ± 0.2	79.6 ± 2.5
NOS^+^/ZnT3^+^	82.6 ± 4.9	73.0 ± 5.2
NPY^+^/ZnT3^+^	87.6 ± 1.3	33.8 ± 3.4
SOM^+^/ZnT3^+^	0	0
VAChT^+^/ZnT3^+^	81.5 ± 4.1	100
VIP^+^/ZnT3^+^	100	100
Thoracic esophagus		
GAL^+^/ZnT3^+^	86.0 ± 6.0	68.0 ± 13.0
NOS^+^/ZnT3^+^	78.0 ± 4.0	92.0 ± 4.1
NPY^+^/ZnT3^+^	33.3 ± 5.2	13.13 ± 6.5
SOM^+^/ZnT3^+^	0	0
VAChT^+^/ZnT3^+^	86.1 ± 4.1	82.9 ± 11.2
VIP^+^/ZnT3^+^	72.0 ± 4.0	95.0 ± 3.0
Abdominal esophagus		
GAL^+^/ZnT3^+^	91.3 ± 3.0	90.3 ± 2.8
NOS^+^/ZnT3^+^	88.8 ± 2.0	95.0 ± 1.5
NPY^+^/ZnT3^+^	95.6 ± 0.5	97.8 ± 2.2
SOM^+^/ZnT3^+^	0	0
VAChT^+^/ZnT3^+^	80.3 ± 4.6	88.3 ± 3.2
VIP^+^/ZnT3^+^	77.9 ± 11.6	87.6 ± 7.5

The numbers of neurons immunoreactive to each substance (at least 500 cells in each “kind” of ganglia of each animal) were considered as representing 100 %

## Discussion

This experiment for the first time shows the presence of ZnT3-LI neurons in the ENS within porcine esophagus, and the large number of such neuronal cells as well as occurrence of them in all types of enteric ganglia strongly suggest important functions of ZnT3 in regulatory processes concerning this part of the GI tract. The obtained results are in agreement with previous studies, where numerous ZnT3-positive neurons have been described in other fragments of porcine digestive tract (Wojtkiewicz et al. 2012a, b). Nonetheless, exact functions of ZnT3 in the enteric nervous system remain unknown. One could only conjecture that these functions can be, at least in part, similar to roles of ZnT3 in the central nervous system, where processes regulated by ZnT3 are better known. Namely, it is well established that ZnT3 is involved in the regulation of zinc levels in neuronal cells, as well as it takes part in transport of zinc into synaptic vesicles (Palmiter et al. 1996). Previous studies described the participation of ZnT3 in both afferent (sensory) and efferent, especially secretory, conduction (Danscher et al. 2001, 2003). Moreover, ZnT3 is known as substance characteristic of inhibitory zinc-enriched nerves (Wang et al. 2002). Other studies showed that this substance is also involved in pathological processes concerning the central nervous system (Takeda 2000; Molnar and Nadler 2001; Kaneko et al. 2015). Since there is a high degree of co-localization of zinc transporter 3 with active zinc in the same neurons of central and peripheral nervous system, ZnT3 is considered to be a substance typical for neuronal cells, which use zinc as the neuromodulator (Wang et al. 2003).

The co-localization of ZnT3 with a wide range of other neuronal active substances, observed both during the present study and previous observations (Wojtkiewicz et al. 2012a, b), seems to support various functions of ZnT3 in the enteric nervous system. It is known that one neuron can contain even several active substances (Furness 2012; Furness et al. 2014), which can be used in different physiological and/or pathological situations, but most frequently, they play similar roles. So, studies on co-localization of Znt3 with other better known substances can be one of possible ways to know the exact functions of ZnT3 in the enteric nervous system. Generally, in the porcine esophagus, ZnT3-positive neurons are also immunoreactive to VaChT (marker of cholinergic neurons), NPY, GAL, VIP, and NOS (marker of nitrergic neurons), which suggest similar functions of ZnT3 and above-mentioned substances. Therefore, short characteristic of their functions seems to be justified.

Acetylcholine is a main neuromediator in the ENS, which first of all stimulates contraction of longitudinal and circular muscles of the GI tract (Porter et al. 1996, 2002), as well as takes part in regulatory processes connected with intestinal excretive activity (Bader and Diener 2015). Important functions of acetylcholine in physiology of the GI tract are confirmed by its presence in various classes of enteric neuronal cells, such as intrinsic primary afferent neurons, ascending and descending enteric interneurons, motoneurons, and intestinofugal afferent neurons (Li and Furness 1998; Dénes and Gábriel 2004; Furness 2012; Chen et al. 2014).

The most of substances observed in ZnT3-positive neurons during the present study such as NPY, VIP, and nitric oxide, in contrast to acetylcholine, are known as strong inhibitory factors impacting on intestinal motility and activity of gut secretion (Nassar et al. 1995; Mourad et al. 2006; Cox 2007; Kasparek et al. 2007). In turn, functions of galanin within the GI tract are not quite clear and depend on animal species and intestinal fragment studied. It is known that GAL shows stimulating influence on the ileal muscles of the rat and pig (Botella et al. 1992), while within the canine pylorus and ileum, it exhibits the relaxatory action (Fox-Threlkeld et al. 1991).

Because the majority of substances observed in ZnT3-positive neurons play inhibitory functions within the GI tract, the obtained results may suggest that zinc transporter 3 in the ENS probably first of all participates in muscular relaxation and processes redacting of excretive activity of mucosal layer. It is in accordance with previous observation in the central nervous system, where inhibitory effects of Znt3 are better known (Danscher et al. 2001). On the other hand, the co-localization of ZnT3 with VAChT suggests that this zinc transporter can also impact the cholinergic stimulatory cells.

Moreover, the present study shows clear differences in the number of ZnT3-positive neurons and in their neurochemical profiles between particular kinds of enteric plexuses as well as the fragment of esophagus studied. This fact strongly suggest that exact roles of this zinc transporter, just like in the case of other enteric neuronal active substances and enteric nervous system as a whole (Fox-Threlkeld et al. 1991; Botella et al. 1992; Furness 2008), clearly depend on the fragment of digestive tract.

To sum up, the obtained results show that ZnT3 is widely distributed in the ENS of porcine esophagus and can co-localize with various other active substances. Probably, it is connected with using of zinc ion as a neuromodulatory factor, but the exact roles of ZnT3 in the enteric nervous system remain not fully explained and require further studies.
